# Supplementary material for: Matching sensor ontologies through siamese neural networks without using reference alignment
Source: PeerJ Comput Sci. 2021 Jun 18;7:e602. doi: 10.7717/peerj-cs.602 (PMC8237319; doi:10.7717/peerj-cs.602)
Supplement: Supplemental Information 1 [file peerj-cs-07-602-s001.zip › 259-4/refalign.html]

# (level 0) Alignment

## Source: http://oaei.ontologymatching.org/2011/benchmarks/101/onto.rdf

## Target: http://oaei.ontologymatching.org/2011/benchmarks/259-4/onto.rdf

## Correspondences

type = type
:   1.0

howPublished = howPublished
:   1.0

periodicity = periodicity
:   1.0

proceedings = proceedings
:   1.0

volume = zsbdgz
:   1.0

annote = annote
:   1.0

PersonList = dsqdbz
:   1.0

month = month
:   1.0

copyright = copyright
:   1.0

Unpublished = Unpublished
:   1.0

address = qzd
:   1.0

Address = Address
:   1.0

chapter = dsazdjz
:   1.0

Chapter = Chapter
:   1.0

editor = dzajj
:   1.0

InBook = InBook
:   1.0

Date = Date
:   1.0

series = series
:   1.0

PageRange = PageRange
:   1.0

date = date
:   1.0

title = dznbaln
:   1.0

Booklet = Booklet
:   1.0

numberOrVolume = numberOrVolume
:   1.0

LectureNotes = LectureNotes
:   1.0

url = url
:   1.0

MastersThesis = xsqlknk
:   1.0

organizer = organizer
:   1.0

mrNumber = mrNumber
:   1.0

pages = pages
:   1.0

TechReport = sdcsqhyz
:   1.0

reviewed = reviewed
:   1.0

startPage = startPage
:   1.0

edition = edition
:   1.0

lccn = lccn
:   1.0

affiliation = affiliation
:   1.0

institution = institution
:   1.0

year = year
:   1.0

isPartOf = isPartOf
:   1.0

organization = organization
:   1.0

country = country
:   1.0

publisher = publisher
:   1.0

school = school
:   1.0

Misc = Misc
:   1.0

collection = collection
:   1.0

Collection = Collection
:   1.0

isbn = isbn
:   1.0

abstract = dsqndsz
:   1.0

directors = directors
:   1.0

Academic = Academic
:   1.0

location = location
:   1.0

MotionPicture = MotionPicture
:   1.0

Article = Article
:   1.0

Informal = qsdsnbsqd
:   1.0

price = dsq
:   1.0

name = name
:   1.0

event = event
:   1.0

state = zdnzadh
:   1.0

Book = Book
:   1.0

book = book
:   1.0

day = day
:   1.0

School = School
:   1.0

shortName = shortName
:   1.0

PhdThesis = PhdThesis
:   1.0

Proceedings = Proceedings
:   1.0

number = number
:   1.0

issue = issue
:   1.0

Reference = sqdsq
:   1.0

endPage = endPage
:   1.0

InCollection = dcsqdcsqd
:   1.0

firstPublished = firstPublished
:   1.0

author = author
:   1.0

Report = Report
:   1.0

note = zdsnsqdv
:   1.0

humanCreator = humanCreator
:   1.0

Conference = zqedzbx
:   1.0

Part = Part
:   1.0

Publisher = Publisher
:   1.0

contract = zandsbh
:   1.0

Manual = Manual
:   1.0

key = key
:   1.0

InProceedings = InProceedings
:   1.0

Journal = Journal
:   1.0

journal = sxqsnbvsq
:   1.0

Monograph = Monograph
:   1.0

keywords = zqdszh
:   1.0

issn = issn
:   1.0

contents = contents
:   1.0

city = city
:   1.0

Institution = Institution
:   1.0

Deliverable = Deliverable
:   1.0

size = size
:   1.0

chapters = chapters
:   1.0

parts = parts
:   1.0

communications = PrSGUs
:   1.0

articles = articles
:   1.0

lastName = lastName
:   1.0

language = language
:   1.0
